# Gap Junctions and Connexins in Microglia-Related Oxidative Stress and Neuroinflammation: Perspectives for Drug Discovery

**DOI:** 10.3390/biom13030505

**Published:** 2023-03-09

**Authors:** Giuseppe Caruso, Lucia Di Pietro, Filippo Caraci

**Affiliations:** 1 Department of Drug and Health Sciences, University of Catania, 95123 Catania, Italy; 2 Unit of Neuropharmacology and Translational Neurosciences, Oasi Research Institute-IRCCS, 94018 Troina, Italy; 3 Scuola Superiore di Catania, University of Catania, 95123 Catania, Italy

**Keywords:** gap junctions, connexins, microglia, oxidative stress, inflammation, carnosine, *N*-acetylcysteine

## Abstract

Microglia represent the immune system of the brain. Their role is central in two phenomena, neuroinflammation and oxidative stress, which are at the roots of different pathologies related to the central nervous system (CNS). In order to maintain the homeostasis of the brain and re-establish the equilibrium after a threatening imbalance, microglia communicate with each other and other cells within the CNS by receiving specific signals through membrane-bound receptors and then releasing neurotrophic factors into either the extracellular *milieu* or directly into the cytoplasm of nearby cells, such as astrocytes and neurons. These last two mechanisms rely on the activity of protein structures that enable the formation of channels in the membrane, namely, connexins and pannexins, that group and form gap junctions, hemichannels, and pannexons. These channels allow the release of gliotransmitters, such as adenosine triphosphate (ATP) and glutamate, together with calcium ion (Ca^2+^), that seem to play a pivotal role in inter-cellular communication. The aim of the present review is focused on the physiology of channel protein complexes and their contribution to neuroinflammatory and oxidative stress-related phenomena, which play a central role in neurodegenerative disorders. We will then discuss how pharmacological modulation of these channels can impact neuroinflammatory phenomena and hypothesize that currently available nutraceuticals, such as carnosine and *N*-acetylcysteine, can modulate the activity of connexins and pannexins in microglial cells and reduce oxidative stress in neurodegenerative disorders.

## 1. Introduction

Microglia can be described as brain-specific macrophages, derived from primitive mesoderm bone marrow precursor cells in the mesodermal yolk sac [1,2]. Microglia represent 10–15% of the glial cells and are a fundamental component of the resident immune system in the central nervous system (CNS) [3].

Current evidence defines “resting” microglial cells as those found in a homeostatic environment [4]. However, the term “resting” is something of a misnomer, because various studies have shown that resting microglia are constantly surveying their surroundings for any molecular signals that could signify an altered state of the environment, such as infection or injury [5]. These “surveillant” microglia have a recognizable ramified morphology characterized by a small soma with almost no cytoplasm from which slim branching processes protrude [6]. While being morphologically uniform at the steady state, microglial physiological responses strongly depend on their surrounding environment and the activity of nearby neurons [7,8,9,10].

When the so-called “surveillant” microglia receive a threatening signal, they shift their phenotype to a reactive “phagocytic” one. This phenotype is characterized by altered metabolic activity, a shift from a ramified to an amoeba-like morphology [11], and increased secretion of cytokines [6]. A similar shift has been observed during aging in the absence of any pathological condition, showing both increased production of pro-inflammatory cytokines and reactive oxygen species (ROS) as well as decreased synthesis of anti-inflammatory and microglial-activation inhibitory factors [12].

Oxidative stress is a detrimental condition caused by the imbalance between the production of ROS and their physiological neutralization [13,14]. ROS formation occurs during the course of normal cell function, such as adenosine triphosphate (ATP) synthesis in the tricarboxylic acid cycle at mitochondria level, but it can be enhanced by noxious stimuli and lead to cellular damage, such as lipid peroxidation, along with the release of pro-inflammatory cytokines that initiate the inflammation cascade [15,16].

In order to counteract the damaging stimuli received, all cells need to communicate with their neighbours to prepare a coordinated response followed by a rescue of the homeostasis. This communication is mediated by the secretion of signalling molecules into the extracellular *milieu*, where they are recognized by other cells and result in a specific molecular response. Many cellular components are in charge of regulating this information flux, such as receptors, vesicles, and junctions [17]. In general, cellular junctions consist of two protein families: connexins (Cxs) and pannexins (Panxs). Cxs are pore structures that allow communication by forming channels that connect directly a cell to an adjacent one or to the environment, while Panxs, similar in structure, are responsible for indirect communication, allowing for the secretion of ions or molecules into the extracellular *milieu* [18].

Cxs, forming gap junctions (GJs) or hemichannels (HCs), and Panxs, forming pannexons, are finely regulated in the physiology of the CNS, and their expression and activity depend on the state of health of the tissue with a specific temporal profile, that can strongly change according to the different pathophysiological states occurring in neurodegenerative disorders [19]. 

In the present review, we will first examine microglial communication and the physiology of channel protein complexes, with a focus on GJs, HCs, and pannexons, in order to understand their contribution to neuroinflammatory and oxidative stress-related phenomena, which play a central role in neurodegenerative disorders. We will then examine how pharmacological modulation of these channels, which are known to be dysregulated in neurodegenerative disorders, can impact neuroinflammatory phenomena. We hypothesize that currently available nutraceuticals, such as carnosine and *N*-acetylcysteine, can modulate the activity of Cxs and Panxs in microglial cells, thus reducing oxidative stress in neurodegenerative disorders, representing a potential additional pharmacological tool to yield neuroprotection in the treatment of these diseases.

## 2. Microglial Cells in Physiology and Pathophysiology: The Role of Inflammation

Microglial cells, as part of the immune system in the CNS, play a pivotal role in maintaining the homeostasis of the brain, and to exert their protective function, they need to be able respond to several different stimuli. The relationship between CNS homeostasis and microglia is bidirectional, in that microglia are both stimulated by any changes in the environment away from homeostasis, but also serve an important role in maintaining the homeostatic environment itself [6]. Moreover, microglia contribute to other fundamental processes, including neurogenesis (during the whole life of the individual [20,21]), oligodendrogenesis [22,23], and brain development. These microglial functions are mediated through the release of trophic factors [24,25] or through the phagocytic activity exerted against apoptotic neurons [20] or synaptic elements in thalamus, cortex, and hippocampus [1,8,26,27,28], the latter being fundamental in the adaptation of brain and behaviours to chronic stress [28].

Microglia are a highly dynamic cell population, constantly remodelling their processes [1,7,29,30,31,32]. According to the evidence discussed above, it is too simplistic to describe them as only mononuclear phagocytes, as was done in the past [33,34,35], because this definition does not include all microglial functions in the CNS. Their classification, previously based on a superficial dichotomic phenotype (pro- or anti-inflammatory), today tends to be specifically based on the stimulus given in a specific cellular and temporal context, with the distinction between phenotypes being made using whole genome expression analysis [36].

Exogenous threatening stimuli (e.g., omega-3 deficiency, bacterial infections, and air pollution) that trigger microglia result in immune activation and a cascade of responses, which can contribute to the impairment of learning, memory, and global cognitive function [37,38] at different stages of life, but are particularly pronounced if they occur during certain critical windows of human development, such as adolescence or during aging [39]. For example, recent studies show that exogenous stimuli, such as lipopolysaccharides (LPS), can have a specific impact on microglia and significantly contribute to the pathophysiology of neurological disorders through the over-expression of pro-inflammatory cytokines, causing oxidative stress, with behavioural consequences, like decreased sociality and locomotor activity in aged rodents [40,41,42,43,44].

Microglia, before being properly activated, undergo priming. This event occurs when microglial cells are exposed to a stimulus, such as the accumulation of aberrant and/or misfolded proteins, like amyloid-β (Aβ) and huntingtin (HTT) during ageing [45], or ROS, or even viral infections. These molecular events increase microglia’s response to a secondary stimulating factor, making them more reactive to milder threatening stimuli [4].

Recently, a new phenotype has been described for microglia in a mouse model [46], morphologically characterized by highly ramified processes that project towards synaptic clefts, encircling axon terminals. These microglia have been called “dark” because of their dark appearance caused by their condensed, electron-dense cytoplasm due to the evident accumulation of oxidative stress-related species. Dark microglia also exhibit other signs characteristic of oxidative stress, such as endoplasmic reticulum (ER) dilatation and mitochondrial disruption [36]. They are also characterized by hyperactivity and have been detected in humans, in the Alzheimer’s disease (AD) brain, as well as in experimental models of chronic stress.

From a translational point of view, perturbed microglial physiology, with regards to the inhibition of phagocytosis, has been found in neurodevelopmental disorders such as a Rett-like syndrome [47] and grooming behaviour (which is the animal model for human trichotillomania) [48], and autism spectrum disorders, as well as in neurodegenerative disorders [49].

## 3. Intercellular Communication in Microglial Cells: Focus on GJs and Cxs

Unlike neurons, microglia are an electrically non-excitable cell population; in the healthy brain, they express few ion channels and have a relatively depolarized resting membrane potential [49]. Even though they do not transmit signals through electrical potential, they communicate effectively with nearby cells by transforming stimuli into a response using an array of membrane-bound receptors that, upon substrate binding, activate specific pathways resulting in the synthesis and release of secondary messengers into the extracellular environment. For the purpose of this review, the terms “sensome” and “secretome”, as they apply to microglia, refer to the whole pattern of transcripts that encode proteins for sensing endogenous ligands and the whole pattern of secreted molecules, respectively. 

### 3.1. Sensome

**Figure 1:**
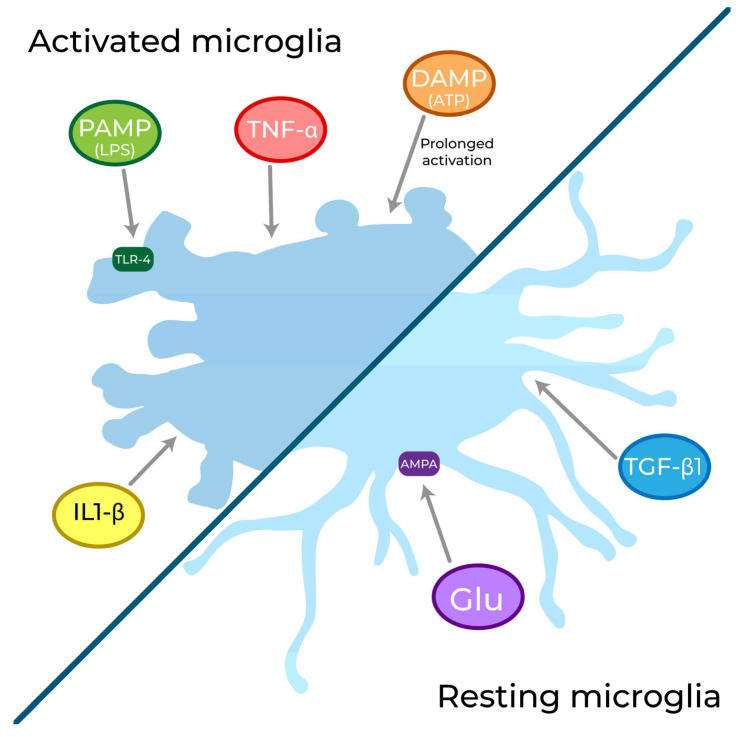
In the CNS, microglial cells are described as the most responsive to external cues. Based on the specific stimulus perceived, they can switch their phenotype from a resting one, characterized by a ramified morphology, to an activated ameboid-like one. Microglia can perceive a variety of the noxious stimuli, including DAMPs (like ATP) and PAMPs (like LPS), as well as pro-inflammatory cytokines like TNF-α and IL-1β. In the resting state, microglia have been shown to express AMPA receptors for glutamate and both of the TGF-β1 receptors that promote their ramified morphology.

Microglia are considered to be more responsive to external cues than any other cells of the CNS. Through the plethora of receptors they express on their surface, they are able to perceive neurotransmitters (through “neural” receptors) and danger signals (through “immune” receptors) [49] (Figure 1).

Concerning neural receptors, microglia express receptors able to respond to the most common neurotransmitters: D1–4 receptors for dopamine, 5-HT2 receptor for serotonin, α1A, α2A, β1, and β2 for adrenaline and noradrenaline, and the α7 nicotinic receptor for acetylcholine [50]. Moreover, they are able to sense bradykinin, endothelin, angiotensin, somatostatin, opioid, neurotrophins, neuropeptides, thrombin, cysteinyl leukotrienes, Notch-1, macrophage colony-stimulating factor, formyl peptide, and lysophosphatidic acid [50]. When considering the pathophysiology of neurodegenerative disorders, glutamate receptors in microglial cells should be considered a primary pharmacological target for their role in excitotoxicity and glutamate clearance under the control of microglia [51]. Glutamate is mainly uptaken by microglia and astrocytes and released through HCs [52]. Among ionotropic glutamatergic receptors, all of the four α-amino-3-hydroxy-5-methyl-4-isoxazolepropionic acid (AMPA) receptor types [53], three of the kainate family [54] and *N*-methyl-D-aspartate (NMDA) are expressed in microglia [55]. On the other hand, metabotropic glutamate receptors belonging to Group I (e.g., mGluR5), Group II (mGluR2,3), and Group III (mGluR4,6,8) coupled to cAMP and regulating tumour necrosis factor-α (TNF-α) release are also strongly expressed [56,57]. Furthermore, receptors for γ-amino butyric acid (GABA) can be found on microglial membranes and play a role in calcium ions (Ca^2+^) signalling and the regulation of K^+^ channels [58].

Regarding receptors related to immune and phagocytic response, microglia express pattern recognition receptors (PRRs) and receptors for tissue mediators. PRRs allow microglia to perceive damage- and pathogen-associated molecular patterns (DAMPs and PAMPs, respectively) and are fundamental to the detection of cellular damage (such as ischemic stroke) and infections [59]. These PRRs are classified into four groups: (1) C-type lectin receptors, mannose, and β-glucan receptors; (2) nucleotide binding and oligomerization domain-like receptors; (3) retinoic acid-inducible gene I (RIG-I)-like receptors, with an RNA helicase domain and two caspase-recruitment domains; and (4) toll-like receptors (TLRs) [60,61,62,63]. These last are widely expressed in glial cells and especially in microglia [64,65,66,67,68]. Among the family of the nine TLRs, TLR-1, -2, -4, -5, and -6 are extracellular, whereas TLR-3, -7, -8, and -9 are intracellular [69]. TLR-1, -2, and -6 are activated by bacterial lipopeptides, lipoteichoic acid, and peptidoglycan. TLR-3 is triggered by virus-specific double-stranded RNA, while TLR-7 and -8 by viral RNA. TLR-4 specifically reacts to LPS and TLR-5 to bacterial flagellin. TLR-9 is instead triggered by bacterial and viral unmethylated CpG DNA [70,71,72,73]. The stimulation of these receptors leads to a cascade of molecular events that is essential to counteract exogenous infections [74].

Microglia are also able to sense inflammatory mediators through the expression of chemokyne and cytokine receptors, such as the receptors for TNF-α, interferons (IFNs), or interleukins (ILs). Among the IL receptors, the IL-1R family, composed of IL-1 type-I receptor (IL-1RI), IL-1 type-II receptor (IL-1RII), and IL-1 receptor accessory protein (IL-1RAcP), plays a central role in the immune response [50]. 

CX3C motif chemokine receptor 1 (CX3CR1) is a unique fractalkine (FKN) receptor [75]. FKN is a transmembrane chemokine expressed in the CNS by neurons, and its receptor is only expressed on microglia [76]. The signal derived from the binding between FKN and its receptor is considered an “OFF” signal, in other words, a signal that contributes in maintaining microglia in a non-activated state [77].

Among the other “OFF” signals that microglia can sense, transforming growth factor-β1 (TGF-β1) plays a central role through two isoforms of the TGF-β receptor, TGF-β-receptor 1 and 2 [78]. TGF-β1 stimulates microglia to adopt a ramified morphology [79] and could be critical for mediating microglial survival and phenotypic differentiation [80].

Finally, ionotropic and metabotropic purinoceptors are also expressed on microglial membranes and are activated by ATP. ATP serves as one of the main messengers of this cell population and is strongly regulated by channel complexes, as will be discussed later in this review. Purinergic receptors are widely expressed in microglia [81]. Ionotropic receptors P2X4 and P2X7 (the latter reflecting the myeloid heritage of microglia [82] generally being expressed in immune cells) have been found. P2X7 is up-regulated under pathological conditions [81,83], contributing to cytokine release and microglial activation [50], which occurs without any exogenous stimuli if P2X7 is overexpressed [84]. P2X7 is involved in Ca^2+^ signalling and not only in ATP sensing, but also in ATP release, as will be discussed later. Expression of P2X4 is induced in the process of microglial activation, especially in the context of neuropathic pain [85]. Metabotropic P2Y2, P2Y6, P2Y12, and P2Y13 purinoceptors, linked to the store-operated Ca^2+^ entry (SOCE) mechanism, have also been found. It has been demonstrated that their prolonged activation has been tied to an uncontrolled Ca^2+^ flux, ultimately leading to microglial activation [86]. Among them, P2Y6, which is sensitive to uridine diphosphate (UDP), controls phagocytosis, while P2Y12, which is activated by adenosine diphosphate (ADP), mediates rapid responses to pathological insults followed by microglial activation [81,87].

As discussed before, the pattern of receptors that a microglial cell expresses strongly depends on the specific environment. For example, under physiological conditions, in a study using intracellular Ca^2+^ signalling as a readout, microglia were found to express P2Y, P2X, and AMPA receptors, but also showed a reduced response to mGluR and acetylcholine receptor agonists, suggesting that they do not express these specific receptors under those conditions [88]. Under other conditions, such as aging, other studies have found that microglia express a reduced pattern of these receptors that are necessary for maintaining normal microglial function [89,90]. Additionally, under inflammatory conditions, such as stimulation with LPS or other inflammatory mediators, microglia also increase expression of K^+^ channels, resulting in enhanced expression of outward K^+^ currents [91,92,93].

It is also worth noting that prolonged activation can make microglia resistant to regulation and impairs their response to mediators, such as IL-10, TGF-β1, and IL-4, as well as to neurons signalling through CX3CL1-CX3CR1 and cluster of differentiation 200 (CD200)-CD200R, with detrimental consequences on the reciprocal interaction between immune and nervous systems [94,95,96,97,98,99,100,101].

### 3.2. Secretome

**Figure 2:**
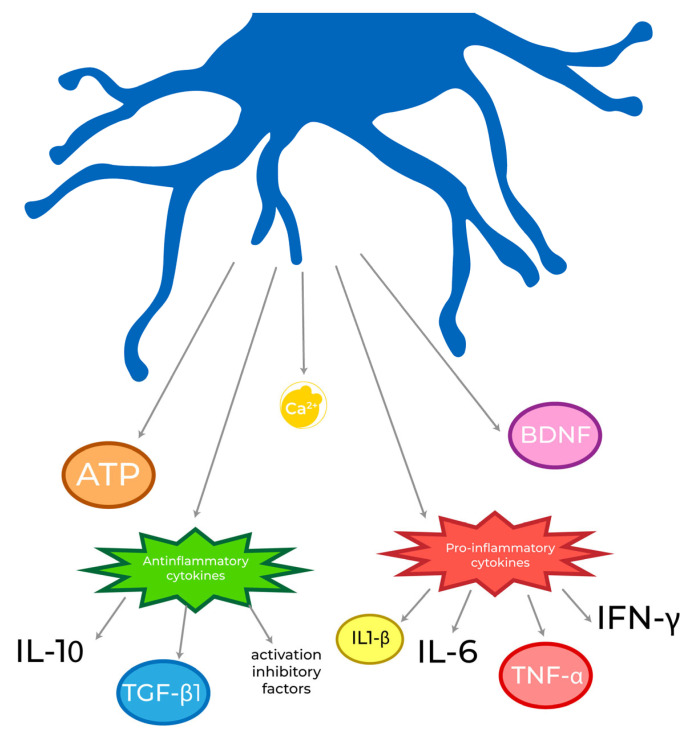
The pattern of secreted molecules that microglia release in the extracellular environment changes according to the health state of the CNS. Microglia are able to release both anti-inflammatory (IL-10, TGF-1β, and inhibitors of activation) and pro-inflammatory (IL-1β, IL-6, TNF-α, and IFN-γ) mediators, together with ATP, BDNF, and Ca^2+^. Through this highly regulated mechanism, they are able to communicate with other cells of the CNS and coordinate a specific response to a dangerous stimulus.

Microglia respond to the detected stimuli by modifying their phenotype from a scanning/ramified one to a phagocytic/ameboid one that allows the clearance of damaged complexes and apoptotic cells from the site of injury [102]. This phenotypic shift also results in a change in the pattern of molecules they secrete (Figure 2).

This altered signal pattern is how microglia communicate specific messages to neighbouring cells. For example, microglia secrete brain-derived neurotrophic factor (BDNF), which in turn has been found to be involved in learning-related synapse formation in the motor cortex [103] as well as neural development [104]. Neurogenesis has also been found to be regulated by microglia in the early postnatal subventricular zone (SVZ) through the release of pro-inflammatory cytokines, such as IL-1β, IL-6, TNF-α, and IFN-γ [105]. The majority of these mediators work by reinforcing microglial activation [106,107].

On the other hand, when found in homeostatic environment, microglia are also able to secrete anti-inflammatory compounds, including IL-10, TGF-β1, as well as microglial activation inhibitory factors, such as CD200 [108].

One of the most important secretion products of microglia is ATP, which acts both as a triggering factor and as an outgoing messenger, activating the purinergic receptors previously described, but also facilitating the communication between microglia and other glial cells, such as astrocytes [109]. ATP and adenosine are released both in physiological synapses [110] and pathological signalling by stressed, damaged, or dying cells [111], representing a classical DAMP.

However, as mentioned above, microglial cells are electrically non-excitable cells, and their pathway of communication, both intra- and extra-cellularly, is mediated by the flux of Ca^2+^, as it is in all glial cells [50,112,113]. This flux regulates both pathological and physiological responses [49]. Ca^2+^ is released by the ER and enters the cells through store-operated Ca^2+^ channels (SOCs) or ligand-gated ion channels. SOC ion channels are Ca^2+^ receptors that are activated when Ca^2+^ is depleted from the ER, and are responsible for SOCE [78]. Ca^2+^ flux in microglial cells is very infrequently registered under physiological conditions [88,114] but increases, either when the anti-inflammatory signalling is interrupted or when a pro-inflammatory trigger stimulates these cells [88,115,116]. These studies suggest that Ca^2+^ flux plays a crucial role in regulating microglial responses and phenotype switching.

### 3.3. GJs, HCs, and Pannexons

When perceiving extracellular threatening stimuli, microglia respond through the secretion of cytokines, communicating the “danger” stimulus to the surrounding cells [117], obtaining synchronization, and ultimately, a better response that increases the probability of restoring the homeostatic environment.

Coupling is obtained through the expression of Cx membrane proteins which group in sets of six to form channels that dock with one another to form a GJ between two adjacent cells, allowing tissue coupling synchronization through direct cytoplasmic connection, both from an electrical and a chemical point of view. This molecular coupling leads to the sharing of ions and gliotransmitters, such as ADP, glucose, lactate, glutamate, cyclic adenosine monophosphate (cAMP), and inositol trisphosphate (IP3)) [118,119]. The channel itself, when not docked, is named HC and is used by the cell to secrete other gliotransmitters (e.g., ATP, glutamate, nicotinamide adenine dinucleotide (NAD^+^), and prostaglandin E2 (PGE2)) into the extracellular environment, allowing the cell to communicate with itself and neighbouring cells via autocrine/paracrine signalling [120]. HCs also allow for the uptake of small molecules (up to ~1–1.2 kDa) and ions (e.g., glucose, cyclic ADP-ribose, (cADPR) and Ca^2+^) [121,122]. 

In humans, Cxs are encoded by twentyone genes [123], each of them corresponding to a different protein isoform. Of the twentyone isoforms, eleven are expressed in the CNS and are found in neurons and glia [124]. From a structural point of view, Cxs consist of four α-helical transmembrane (TSM) domains (M1–M4) connected by one cytoplasmic loop and two extracellular loops (E1–E2), with both the N-terminus and C-terminus in the intracellular domain [18]. Cxs are highly conserved proteins, especially throughout the TSM domains and extracellular loops, while the cytoplasmic loop and the C-terminus can show great variation in both sequence and length [18], with many sites susceptible to phosphorylation and other post-translational modifications that influence the whole (structure and function) channel physiology [125].

As mentioned before, six Cxs bind together to form a HC: this can be homomeric if made up of all the same Cxs or heteromeric if formed by the combination of different Cx subunits. Five different isoforms of Cxs (each named after their weight expressed in kDa) have been found to be expressed in microglia, depending on the state in which the cell is found; Cx32 and Cx36 are mainly expressed under a physiological “resting surveillance” state, while the Cx43 mRNA level is increased in activated microglia [126,127,128]. Cx46 and Cx29 have also been found on cell membranes of activated microglial cells [129,130].

Microglia are not only able to form functional GJs among themselves, but also with hippocampal neurons (using Cx36) [131,132] and neuronal precursor cells (NPCs) at the level of SVZ [133,134]. Microglial-NPC GJ communication was found to be fundamental in sustaining the reciprocal signalling necessary for the proper development of neurogenic niches [134]. The ability of Cx32 to form HCs has been well-characterized and is known to release glutamate following TNF-α stimulation [135].

**Figure 3:**
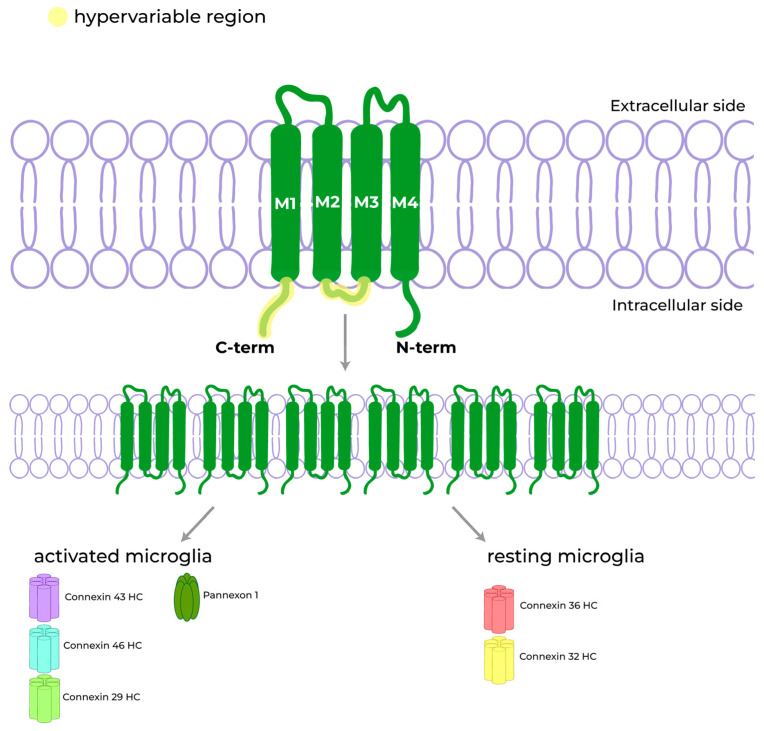
Cxs are made up of four transmembrane alpha helical domains (M1–M4), two extra-cytoplasmatic loops, and three intracellular domains. Cxs are highly conserved proteins except for the C-terminus and the intracellular loop, which are subject to variations. Six Cxs form a single HC, and different HCs are expressed by microglia according to their activation status: Cx43, Cx46, and Cx29 are typical of activated microglia, while Cx36 and Cx32 are mostly found in resting microglial cells. Panxs are similar to Cxs in structure and composition. The most represented one in microglia is Panx1, that assembles a hexamer in a manner similar to Cxs.

Pannexons are another class of molecular structures involved in cellular communication and are similar in organization and function to connexons. They are TSM channels composed of six Panx subunits (except for Panx2 which forms octamers [136]). Pannexons are permeable to molecules below 900 Da, such as nucleotides and ions [137]. Of the three different isoforms of Panxs found in mammals (Panx1, Panx2, and Panx3), only two (Panx1 and Panx2) are widely expressed in the CNS [138], and only Panx1 is expressed in microglia in the resting surveillance state [139] (Figure 3).

Moreover, under normal physiological conditions, microglial HCs and pannexons are preferentially closed [19]. Their gating is regulated by different factors: HCs and GJs can open or close due to changes in the TSM potential or Ca^2+^ concentrations, mechanical stimulation, chemical changes such as phosphorylation or nitrosylation, and in response to ROS [129], while pannexons are strongly involved in the response to inflammatory and excitotoxic insults [129]. On the other hand, GJs are open under normal physiological conditions and can often be closed or down-regulated under pathological conditions [140].

## 4. Role of GJs, HCs, and Pannexons in Oxidative Stress and Neuroinflammation

Oxidative stress can be broadly defined as an imbalance between the physiological production of reactive species and their neutralization through intracellular antioxidant mechanisms [141,142]. Oxidative stress can be due to the overproduction of different chemical species including ROS, such as superoxide anion (O_2_^−•^) and hydroxyl radicals (•OH), as well as reactive nitrogen species (RNS), such as nitric oxide (NO) and peroxynitrite (ONOO^−^) [141,143,144,145]. ROS often enhance a cell’s inflammatory processes by interacting with cellular organelles and interfering with physiological machinery [146], thus leading to changes in cellular behaviour, resulting in changes in both the transcriptome and the proteome [147].

Neuroinflammation can be defined as an innate immunological response of the nervous system involving microglia, astrocytes, cytokines, and chemokines, which plays a central role in an early phase of neurodegenerative disorders such as AD [148,149]. During neuroinflammation, the brain tries to recover from a triggering stimulus given by exogenous (e.g., bacterial infection) or endogenous (e.g., misfolded protein) cues. This response is aimed to restore the homeostasis of the nervous tissue, but a prolonged overactivation of this response has detrimental effects on neural cells, eventually causing their death [150].

Glial cells are the main actors in these processes [150], and microglia, in particular, are the cells tasked with responding to threatening stimuli, because they are the primary component of the immune system in the CNS [151]. 

Pro-inflammatory signals, communicated via GJs, HCs, and pannexons, recruit resting microglia toward the injury site and cause them to switch their phenotype and become activated. In addition, a pan-glial network has been described, involving non-microglial cells with the aim of repairing the damaged tissue to restore the homeostatic environment [152]. Microglial function is also being hypothesized to favour cross-presentation of antigens at the immune synapse [19], based on the evidence that these cells are the main antigen presenting cells (APCs) in the CNS [153] and that GJs are used by other APCs, such as dendritic cells, to transfer antigens [154,155]. 

However, while GJs, HCs, and pannexons are crucial in restoring the homeostasis in response to threatening stimuli in the CNS, over-activation of these communication channels can prove detrimental to neuronal survival. In particular, the non-specific opening of HCs and pannexons, which alters the permeability properties of the nervous tissue, can promote the onset and progression of different neurodegenerative diseases [156,157,158].

### 4.1. Microglial GJs, HCs, and Pannexons Modulation in Inflammation and Oxidative Stress

Microglial coupling is enhanced by cytokines and exposure to bacterial-derived agents [19], contributing to inflammation in CNS. After the shift from a resting state to the activated phenotype, many Cxs are up-regulated (e.g., Cx29 [130], Cx32 [130,135,159], Cx36 [160], and Cx43 [133,139,161,162,163,164]).

The functional modulation of HCs, pannexons, and GJs, namely, Cx43 HCs and Panx1 channels opening allowing the release of ATP, is due to the activation of the inflammasome complex and the nuclear factor kappa-light-chain-enhancer of activated B cells (NF-κβ) pathways, which release pro-inflammatory cytokines (e.g., IL-1β and TNF-α) in response to different PAMPs, such as advanced glycation end products (AGEs) and LPS, and DAMPs that activate PRRs [19,165]. The activation of NF-κβ pathway has been related not only to inflammation but also to oxidative stress [166].

Specifically, AGEs activate their receptors (RAGEs), LPS triggers the TLR-4 [167], and *Staphylococcus aureus* derived peptidoglycan (PGN) triggers the TLR-2 [168]. The NF-κβ pathway activation, on which the three receptors triggering converge, activates two different autocrine/paracrine releases: (1) IL-1β, acting on its receptor as well as on accessory proteins (IL1RI and IL1RAcP), leads to ATP release through Cx43 HCs and Panx1 channels; (2) TNF-α, acting on its receptor TNFR1, leads to the activation of glutaminase and to the release of glutamate in a dose-dependent manner and proportional to the number of Cx32 HCs expressed, converging on excitotoxicity and neuronal death [135]. Both IL-1β and TNF-α, being the effectors of the cascade starting from NF-κβ, are well-known GJ function inducers in microglia [127,139,161]. TNF-α contributes to microglial activation and communication in a way that is dependent on Cx43, increasing its expression and microglial coupling when administered with IFN-γ/ATP or when triggered by PGN (as demonstrated through the use of lucifer yellow (LY)) or, even, together with HCs opening when stimulated by AGEs [127,139,161,164]. According to this scenario, concerning channel complexes, Cx43 and Panx1 represent the main target of inflammatory cascade [139]. 

**Figure 4:**
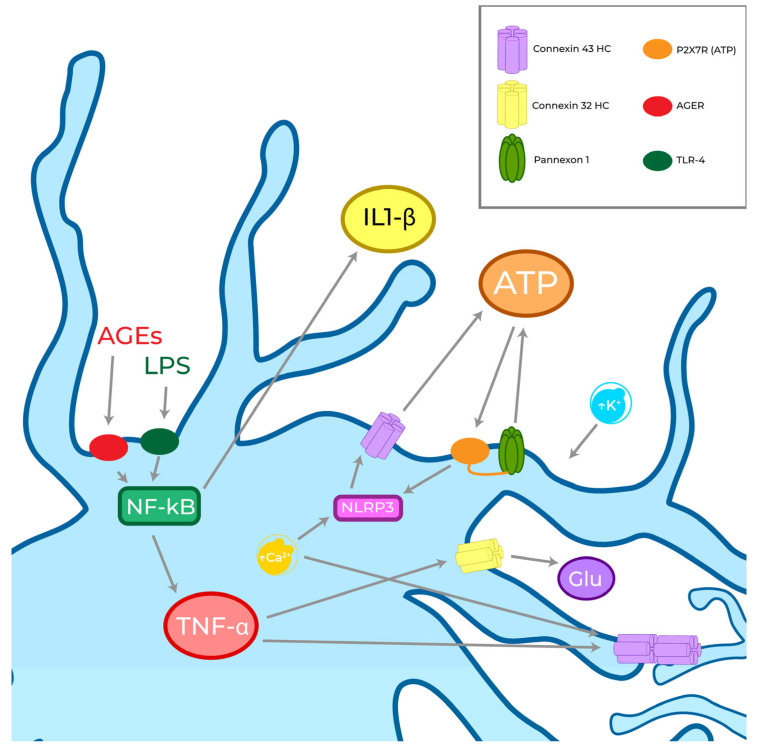
Cxs are fundamental in microglial communication, specifically because of their ability to secrete ATP and glutamate, known to be messengers of danger in the extracellular environment. Their gating is highly regulated by the inflammation pathway (NF-κB) through the release of TNF-α. ATP acts as an autocrine messenger and sustains its own release by activating NLRP3 pathway. It worth noting that a physical interaction between P2X7R and Panx1 has been observed.

The release of ATP in the extracellular environment, due to the stimulation promoted by TNF-α and ATP, leads to an increase in intracellular Ca^2+^ concentrations ([Ca^2+^]_i_) and the activation of P2X receptors via autocrine signalling, and consequently increases the release of IL-1β followed by up-regulation of GJs [139]. At the same time, LPS-induced ATP release was suppressed by the inhibition of Panx1 channels [169], and was instead activated by the increase in extracellular K^+^ concentration [170]. ATP is recognized by the P2X7 receptor as a DAMP derived from damaged cells and causes inflammasome activation [171]. Additional evidence confirms this direct relationship between P2X7R ATP-sensing and ATP release by Panx1 channels. In fact, the Panx1 channel is able to release ATP through a protein–protein interaction with P2X7R [172]. This interaction was shown by the coimmunoprecipitation of Panx1 with P2X7Rs [173,174,175,176,177] and demonstrated that the interaction mechanism involves proline 451 in the C-terminal tails of these receptors [178,179]. P2X7R is also able to open Cx43 HCs [121,180,181,182]. Additionally, P2YRs are related to ATP secretion in microglia by enhancing Ca^2+^ release that induces HCs and pannexons [183,184,185]. Therefore, ATP release is facilitated by Cx43 HCs and Panx1 channels, and this relationship has been demonstrated after stimulation with LPS [186], Aβ [186], or ZnCl_2_ [187,188] (Figure 4). 

Another messenger with a primary role in this context is glutamate, whose increased release has been detected from glial cells during TNF-α-induced inflammation, as discussed above. These molecular events involve glutamate receptors (NMDA, AMPA, and mGluR), in particular, extrasynaptic NMDA receptors in neurons of the CA1 region of the hippocampus, ultimately leading to excitotoxicity and neuronal death [189]. The increased excitability of pyramidal neurons is also related to the propagation of Ca^2+^ waves from astrocytes [190], which also positively regulates neuroinflammation [191]. This increased neuronal firing leads to extracellular pH changes that sustain inflammation, hyperexcitability, and synchronicity, ultimately leading to uncontrolled opening of these channels that maintains the pathological condition [124]. In addition, the release of gliotransmitters modulates the inflammatory response, activate immunological cells, and causes irreversible neuronal damage [192].

Different experimental models of CNS disorders have contributed to the clarification of this scenario. Prenatal nicotine exposure followed by a high-fat diet in offspring mice, led to an increased opening of Cx43 HCs in astrocytes and Panx1 pannexons in microglia and neurons. The above-mentioned events are differentially inhibited by the blockage of inducible nitric oxide synthase (iNOS), cyclooxygenase 2 (COX2), prostaglandin E receptor 1 (EP1), P2X7, and antagonism of NMDA receptors, that in turn reduces ATP and glutamate-detrimental release in the hippocampal slices from offspring mice. Starting from this evidence, the authors hypothesized that channel complexes are involved in brain alterations in offspring, resulting from mothers smoking tobacco during pregnancy [193].

The role of Ca^2+^ flux in regulating the gating of HCs in microglial cells has been also demonstrated. The increase in [Ca^2+^]_i_ has been linked to an up-regulation of Cx43, combined with an enhanced LY coupling [194]. This coupling induces an intracellular Ca^2+^ wave that spreads through microglial cells and significantly augments glutamate release (through HCs and Pannexons). This stimulates NMDA receptors in neurons that end up increasing their Cx36 HCs and Panx1 channels activity, with a reduction of cell viability [162]. This regulation further leads to the release of Ca^2+^, causing imbalances in Ca^2+^ flux and finally, provoking neuronal death. Moreover, Ca^2+^ flux, and the resulting Ca^2+^ waves, are known to be increased by noxious stimuli and redox disequilibrium in both the inflammatory state and during the activation of purinergic P2X receptors [195]. In hippocampal neurons, Ca^2+^ influx also stimulates Panx1 channel activity [196], but no studies have been conducted in microglial cells as of yet. 

In the context of this self-sustaining and detrimental inflammatory communication, P2X receptors are activated by ATP, whose release is increased by the opening of HCs and Panx1 pannexons. Along this line, the synergistic activity of Ca^2+^ waves, the enhanced release of ATP in the inflammatory pathway, acting on HCs and pannexons, and the activation of NMDA and P2X7 receptors observed in neurons were shown to be linked to the increased release of ATP from microglia [162]. This hypothesis has been validated by showing that the inhibition of Cx43 and Panx1 channels completely prevented this uncontrolled release [197]. The P2X7R, together with P2X4R, exerts its function through the activation of the pyrin domain-containing-3 (NLRP3) inflammasome, enhancing the inflammatory effects through the up-regulation of Cx43 expression [198]. P2X7R activation also increases [Ca^2+^]_i_ [199]. To summarise, microglial-enhanced Ca^2+^ waves spread through GJs and ATP release via HCs promote neuronal death. 

Moreover, Ca^2+^ signalling has a significant role in regulating ROS homeostasis. It is known that oxidative stress is increased by Ca^2+^ influx [200,201] as well as extracellular ATP and was demonstrated in both skeletal muscles (through P2Y1) [202] and hippocampal neurons [203]. In this scenario, ATP regulates Ca^2+^ influx [204] through the stimulation of P2Y2 receptors with the involvement of Panx1 [205,206]. These molecular events start from the enhanced ATP release that leads to the activation of Panx channels and increased Ca^2+^ influx with a commensurate increase in the production of ROS and the activation of the previously mentioned NLRP3 inflammasome [207]. Together with this evidence, the direct involvement of Panx1 into the NLRP3 pathway has been demonstrated [174,208], thus generating a neurobiological link between oxidative stress and inflammatory cascades involving channel proteins.

While this evidence does provide a clear link between oxidative stress and channel protein regulation, further studies are needed to assess the scope of microglial involvement in the above-described processes.

### 4.2. The Interaction between Reactive Microglia and Neural Cells in CNS: Role of Connexins and Pannexins

**Figure 5:**
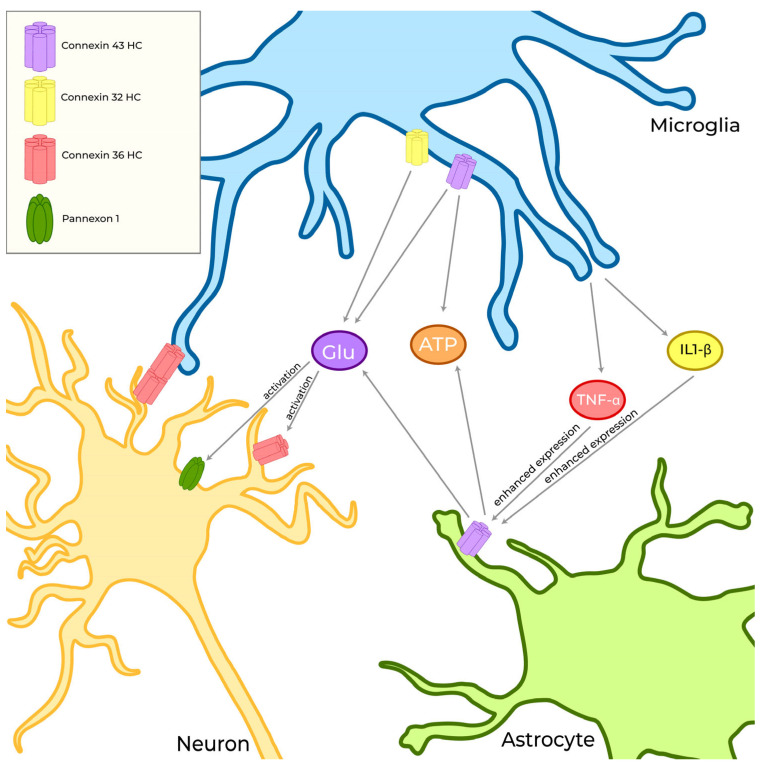
Cxs expressed by microglia are fundamental for their interaction with other cells of the CNS. This interaction occurs both indirectly, through the release of metabolites such as glutamate (Glu), ATP, TNF-α, and IL1-β that can up-regulate or modulate HCs in neurons and astrocytes, and directly, involving the formation of Cx36 GJs between microglia and neurons.

As discussed, microglia can communicate with themselves using GJs, HCs, and pannexons, but can also communicate bidirectionally with astrocytes, neurons, and oligodendrocytes through channel proteins and complexes. Herein, we present a brief analysis of the relationships that exists between microglial and neural cells involving Cxs and Panxs (Figure 5).

#### 4.2.1. Astrocytes

Activated microglial cells communicate the detection of a threatening stimulus to astroglia through the secretion of pro-inflammatory cytokines, such as TNF-α and IL-1β [209]. These cytokines enhance Cx43 expression while reducing GJ functions, thus demonstrating that HCs and GJs have a different and opposite regulation in response to an inflammatory stimulus [210,211]. On the other hand, it has been demonstrated that microglial depletion leads to decreased coupling in the astrocytic network due to, among other causes, the down-regulation of Cx30 and Cx43 [212].

Astrocytes, in turn, have an immunomodulatory action over microglia [213,214] that involves the spread of Ca^2+^ waves occurring through the ATP-induced ATP release [215,216]. One of the mechanisms underlying this phenomenon involves the release of ATP through Cx43 that acts on purinergic receptors expressed on microglia and has a downstream effect of increasing glutamate release, through Cx43-Cx32 HCs, and inducing neuronal damage [19].

During an inflammatory event, astrocytes are also able to secrete anti-inflammatory factors, such as TGF-β1, in order to suppress the self-perpetuating release of ATP by microglia [186]. Moreover, the release of ATP and glutamate by astroglial Cx43 HCs [217] suggests that astroglia contribute to the activation of microglia and the resulting channel modulation of neuronal Panx1 channels. 

#### 4.2.2. Neurons

In the CNS microglia significantly contribute to the maintenance of neuronal homeostasis and neural transmission. As a result, a dysregulation of microglial activation has a significant impact on neuronal survival [218]. The detrimental effect on neurons by ATP and glutamate release from microglia (also occurring through HCs as previously discussed) is significantly diminished in Cx43 knock-out mice, suggesting that microglia (but also astrocytes) induce neuronal death when stimulated by Aβ by triggering HCs in neurons [162]. Moreover, microglial release of glutamate and ATP contributes to the activation of Panx1 channels and Cx36 HCs in neurons, leading to unbalanced Ca^2+^ flux and increased oxidative stress [129,219].

#### 4.2.3. Oligodendrocytes

Oligodendrocytes play a role in tissue synchronization by expressing GJs formed by Cx47 and creating both homotypic (oligodendrocyte–oligodendrocyte) and heterotypic (oligodendrocyte–astrocyte; Cx47-Cx43) channels [220]. However, conflicting data have been found regarding the presence of GJs between oligodendrocytes and astrocytes [221], and further studies are needed to better assess the presence of this type of GJ as well as the existence of a direct coupling between oligodendrocytes and microglia.

## 5. Protein Channel Modulation in the Treatment of Neurodegenerative Diseases: Perspectives for Drug Development

The implications of microglial cells in spreading inflammatory signals throughout the CNS through GJs, HCs, and pannexons, as well as the role of channel complexes in promoting neuronal death, have been deeply discussed above. Therefore, we can hypothesize that protein channel modulation/inhibition could represent a novel pharmacological strategy to counteract neurodegenerative phenomena that arise from the modulation of inflammatory signalling [222].

**Figure 6:**
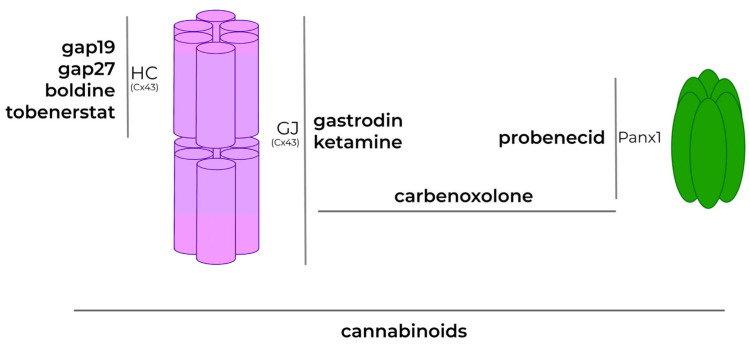
Cxs as potential pharmacological targets. Several compounds with a modulating effect to different targets were proposed: gap19, gap27, boldine, and tobenerstar for HCs; gastrodin and ketamine for GJs; probenecid for Panx1 channels. Carbenoxolone acts on both Panx1 channels and GJs, while CBs acts on Cx38, Cx26, and Cx43.

Cxs have already been shown to be involved in the pathophysiology of neurodegenerative diseases [223]. Modification of physiological functioning and expression of Cxs- and Panxs-based channels have been found in neurological diseases, such as AD and Parkinson’s disease (PD). In fact, the accumulation of Aβ plaques and α-synuclein, the clinical hallmarks of AD and PD, respectively, are associated with increased levels of Cx43 and chronic activation of the HCs they form. In particular, concerning AD, increased Cx43 HC activity has been shown to occur in microglia, neurons, and astrocytes after stimulation with Aβ oligomers, known to be the most toxic species of Aβ aggregates [162,224]. Moreover, in Huntington’s disease, increased Cx43 GJs have been related to the mitochondrial fragmentation deriving from the abnormally long polyglutamine found in the HTT protein [223]. Based on the above, Cxs and Panxs have been investigated as potential therapeutic targets in neurodegenerative disorders [225]. However, the limitation in the drug discovery processes stems from the fact that Cxs and Panxs are widely expressed in the CNS obscuring different physiological roles in strict dependence on their localization. As a result, it is challenging to develop molecules that target a specific protein isoform or specific structures (i.e., HCs or GJs) that do not also interfere with the normal physiological functions of necessary channel complexes [226]. Despite this, many molecules are under investigation based on their known ability to serve as GJ blockers [226] and some have demonstrated at least a partial preclinical efficacy in the treatment of neurodegenerative disorders. In particular, gap19, a Cx43 blocker, reverted dopaminergic neurons loss in a murine model of PD and extinguished microglial activation [227]. Concerning AD, the alkaloid boldine (derived from the boldo tree *Peumus boldus*) has demonstrated a selective pharmacological activity in inhibiting astrocytic and microglial HCs, without affecting GJs. Its activity was tested in a murine model of AD and led to a reduction of ATP and glutamate release in the hippocampus, resulting in reduced neuronal degeneration [225,228]. Additionally, cannabinoids (CBs) have been shown to regulate the opening of GJs, HCs, and pannexons during neuroinflammation [217,229], particularly in neurons [205] and astrocytes-altered networks [230,231,232]. CBs are also able to inhibit Cx43 function, and the release of pro-inflammatory cytokines from microglial cells in pro-inflammatory conditions, thus sustaining neuronal survival [233]. Probenecid, an inhibitor of organic anion transporter, acts on Panx1 by blocking the channel in a concentration-dependent manner, probably through interacting with its extracellular loop (a mechanism of action that is shared with CBs towards HCs) [234]. Many other GJ blockers have been tested with positive results on the modulation of CNS channels in experimental models of neurological disorders, even if a direct action on microglial cells has not been demonstrated: (i) gap27 inhibits Cx43 in the treatment of neuropathic pain induced by the antineoplastic drug vincristine [235]; (ii) carbenoxolone suppresses the activity of Panx1, Panx2, P2X7, Cx38, Cx26, and Cx43 GJs [236,237,238], the last of which being involved in the rescue from α-synuclein-induced inflammation in a PD model [239]; (iii) tonabersat, together with its recognized effects in down-regulating Cx26 expression, diminished ATP release from Cx43 HCs in a preclinical model of ischemia and has been proposed for the treatment of migraine [238]; (iv) ketamine, and other anaesthetic drugs, inhibit GJs formation between astrocytes in vitro [240]. Additionally, the naturally derived gastrodin has shown positive effects concurrently inhibiting Cx43 GJ formation and phosphorylation and counteracting PD-like phenotypes [241,242] (Figure 6).

Recent evidence suggests that specific natural compounds and nutraceuticals, such as carnosine and *N*-acetylcysteine, can modulate the activity of Cxs and Panxs and are examined in the following paragraphs for their therapeutic potential in neurodegenerative disorders.

### 5.1. Carnosine

Carnosine is an endogenous dipeptide formed by the ligation of β-alanine and L-histidine performed by the carnosine synthase 1 (CARNS1) enzyme, especially in tissues with an increased oxidative metabolism (e.g., skeletal and cardiac muscles and CNS) [243]. In the CNS, it is synthetized by oligodendrocytes, but it acts on microglia, astrocytes, and neurons [244,245]. Carnosine’s imidazole ring is responsible for its activity as a ROS scavenger, functioning as an antioxidant [9]. Moreover, one of its derivatives, 2-oxo-carnosine, is produced by neuroblastoma cells (SH-SY5Y) and seems to have a stronger antioxidant potential when compared to other detoxifying cell machineries [246,247]. One of the recently identified potential pathways through which carnosine is expected to exert its neuroprotective and antioxidant function is the nuclear factor erythroid 2-related factor 2 (Nrf2) pathway, which is itself the main regulator of cellular antioxidant response [248,249,250,251]. The anti-inflammatory activity of carnosine has also been linked to the regulation of the release of pro-inflammatory mediators along with its ability to chelate metal ions such as Zn^2+^ [245]. Polaprezinc, a complex formed by carnosine and zinc, is able to inhibit the NF-κB signalling, reducing IL-8 release and inducing heat shock protein 72 (Hsp72) [252,253,254]. The chelation of Zn^2+^ and Cu^2+^ by carnosine has also been linked to the regulation of NMDA-mediated glutamate transmission along with a fine regulation of the synaptic impulse [245,253]. Moreover, carnosine is able to decrease oxidative stress-inducing species (e.g., NO and O_2_^−•^), enzymes (i.e., iNOS and NADPH oxidase enzymes), as well as the expression of pro-inflammatory cytokines, such as IL-1β [255,256]. It should be also taken into consideration that carnosine can exhibit neuroprotection through the modulation of immune cells, such as macrophages and microglia [257,258], by modulating their polarization and ameliorating dysregulated cellular energy metabolism [257,259].

When considering the anti-inflammatory and antioxidant activity of carnosine in the CNS [260,261], it becomes particularly interesting to examine if its multimodal pharmacodynamic profile also includes the regulation of Cxs and Panxs. This hypothesis starts from the evidence that carnosine has demonstrated a relevant preclinical efficacy in experimental models of neurodegenerative diseases such as AD and PD, characterized by elevated oxidative stress and inflammation [244,262,263,264,265,266] and strongly linked to channel protein complexes dysregulation. Along this line, carnosine is known to modulate factors that are released through the up-cited channels, such as glutamate, which is released through Cx32 HCs [135]. Glutamate transporter-1 is regulated by carnosine and, thus, can then reduce extracellular glutamate concentrations [267,268]. It is also worth noting that carnosine has shown promise in the treatment of cardiovascular-related pathologies. For example, carnosine improved myocardial contractility in isolated rat hearts by regulating [Ca^2+^]_i_, demonstrating its potential use for treating contractile failure commonly associated with ischemic heart disease [269]. Additionally, carnosine was shown to attenuate the vascular calcification process, prevalent in the aging population, in an in vitro model in a dose-dependent manner [270]. Together with this evidence, carnosine is also known to increase IL-10 and TGF-β1 levels [255]. Carnosine-induced TGF-β1 release from microglia could therefore interrupt the aberrant and self-perpetuating ATP release mediated by the channels overactivation [186]. Further studies are needed to better understand the impact of carnosine-induced TGF-β1 in the modulation of Cxs and Panxs activity.

### 5.2. N-Acetyl Cysteine (NAC)

NAC is the *N*-acetyl derivative of the natural amino acid L-cysteine [271]. NAC acts as an antioxidant either directly or indirectly as a reduced glutathione (a well-known antioxidant) precursor. 

Different studies show that the antioxidant effects of NAC can interfere with ATP release. In particular, it has been demonstrated that NAC counteracts the effects of the inhibition of the ectoenzyme CD38 by 8-Br-cADPR, a ryanodine receptor antagonist, whose action led to increased release of ATP and opening of Cx43 HCs [272]. In addition, the authors found that the natural compound 18-α-glycyrrhetinic acid acts as a hemichannel blocker causing a diminished opening of the channel, known to be related to the occurrence of oxidative stress. Along this line, NAC intervention was implemented in a model for traumatic brain injury and was found to reduce oxidative stress and concomitantly, the expression of Cx40 that is strongly related to it [273]. We can then hypothesize that NAC can modulate oxidative stress-related expression of HCs, although the impact of this compound in the modulation of channel proteins expressed in microglial cells remains unexplored.

## 6. Conclusions and Future Perspectives

The roles of microglial GJs and HCs in mediating oxidative stress and neuroinflammation are emerging as central factors in the pathophysiology of neurodegenerative disorders. The extracellular release of ATP and glutamate gliotransmitters, together with Ca^2+^, seems to exert a pivotal role as messengers of neuroinflammation to coordinate a synchronized response in the CNS, allowing for a better and more probable restoration of neuronal homeostasis after a threatening stimulus. Nevertheless, further studies are needed to better comprehend the exact roles and molecular mechanisms underlying the complex network of neuroinflammation and oxidative stress involving channel proteins. Considering the key role of these detrimental phenomena in the progress of neurodegenerative diseases, future preclinical studies should explore the neuroprotective activity of recently identified HC inhibitors as well the neuroprotective potential of carnosine and NAC in experimental models of AD and PD.

## Data Availability

Not applicable.
